# Broad-Scale Patterns of Late Jurassic Dinosaur Paleoecology

**DOI:** 10.1371/journal.pone.0012553

**Published:** 2010-09-03

**Authors:** Christopher R. Noto, Ari Grossman

**Affiliations:** 1 Department of Biomedical Sciences, Grand Valley State University, Allendale, Michigan, United States of America; 2 Department of Anatomy, Midwestern University, Glendale, Arizona, United States of America; University of Zurich, Switzerland

## Abstract

**Background:**

There have been numerous studies on dinosaur biogeographic distribution patterns. However, these distribution data have not yet been applied to ecological questions. Ecological studies of dinosaurs have tended to focus on reconstructing individual taxa, usually through comparisons to modern analogs. Fewer studies have sought to determine if the ecological structure of fossil assemblages is preserved and, if so, how dinosaur communities varied. Climate is a major component driving differences between communities. If the ecological structure of a fossil locality is preserved, we expect that dinosaur assemblages from similar environments will share a similar ecological structure.

**Methodology/Principal Findings:**

This study applies Ecological Structure Analysis (ESA) to a dataset of 100+ dinosaur taxa arranged into twelve composite fossil assemblages from around the world. Each assemblage was assigned a climate zone (biome) based on its location. Dinosaur taxa were placed into ecomorphological categories. The proportion of each category creates an ecological profile for the assemblage, which were compared using cluster and principal components analyses. Assemblages grouped according to biome, with most coming from arid or semi-arid/seasonal climates. Differences between assemblages are tied to the proportion of large high-browsing vs. small ground-foraging herbivores, which separates arid from semi-arid and moister environments, respectively. However, the effects of historical, taphonomic, and other environmental factors are still evident.

**Conclusions/Significance:**

This study is the first to show that the general ecological structure of Late Jurassic dinosaur assemblages is preserved at large scales and can be assessed quantitatively. Despite a broad similarity of climatic conditions, a degree of ecological variation is observed between assemblages, from arid to moist. Taxonomic differences between Asia and the other regions demonstrate at least one case of ecosystem convergence. The proportion of different ecomorphs, which reflects the prevailing climatic and environmental conditions present during fossil deposition, may therefore be used to differentiate Late Jurassic dinosaur fossil assemblages. This method is broadly applicable to different taxa and times, allowing one to address questions of evolutionary, biogeographic, and climatic importance.

## Introduction

Over the past twenty years new fossil discoveries, novel technologies, and a proliferation of analytical techniques have greatly increased our knowledge of dinosaur morphology, phylogeny, and behavior. These data, when combined with our growing knowledge of dinosaur biogeography, not only make it possible to address complex questions about changing dinosaur distributions, but also broad-scale ecological questions about the nature of dinosaur-dominated communities.

Advances in computer software enabled the creation of large, accessible databases recording data from an ever-increasing number of localities as new discoveries are made [1]–[3]. Coupled with geographic information system (GIS) technology, this allows us to examine patterns of dinosaur distribution at broad–regional to global–scales. There have been numerous studies of this type on dinosaur biogeography, focusing on vicariance, dispersal, and extinction patterns [4]–[10], which have helped identify possible areas of endemism, directions of dispersal, and even test the validity of different paleogeographic reconstructions. However, these data and methods have not yet been applied to ecological questions.

Historically, the ecology and behavior of dinosaurs has been reconstructed with reference to modern analogs, living animals that contain similar physical attributes or are closely related. Depending on the researcher, the modern analogs used to describe dinosaur ecology and behaviors have ranged from crocodilians and birds to mammals [11]–[21]. These models for dinosaur ecology and behavior are valuable starting points, providing a necessary conceptual framework from which to evaluate the unusual morphology of these extinct creatures. In many ways such thinking has been highly informative, as it is impossible to explain the biology of extinct taxa without first studying how living organisms operate. On the other hand it may lead to erroneous or unrealistic reconstructions based on constraints imposed by the organismal model used in the analogy, especially when those comparisons exist in the absence of quantitative data. Also, the focus on reconstructing the ecology of particular dinosaur taxa has hampered exploration of dinosaur-dominated ecosystems as a whole. While understanding the ecology of individual taxa is important, this information is insufficient outside of a broader ecosystem context.

An excellent example of a whole-ecosystem study is that of Foster [22], who carried out a detailed ecological analysis of the Late Jurassic Morrison Formation in the western United States. Such studies are few due to the immense effort involved in bringing together the multiple lines of data necessary to carry out such a comprehensive analysis. Therefore, detailed work of this nature is currently lacking for the large number of dinosaur-fossil bearing formations around the world. To gain a broad-scale view of dinosaur-dominated ecosystems a different approach is needed.

An ecological approach that reconstructs general habitat characteristics and ecological diversity among dinosaur faunas (as interpreted from fossil assemblages) in different regions of the globe is an important tool for understanding some of the many forces shaping dinosaur distribution patterns. In addition, deciphering changes in the ecological structure of dinosaur communities over time is a powerful tool for elucidating the role of global changes in climate, continental arrangement, and land area in shaping dinosaur biogeography, ecology, and evolution. Various dynamics, such as Milankovich cycles (which impact solar energy distribution across the Earth's surface), or plate tectonics (which largely determine the location, size, and geology of terrestrial landmasses), affect climate patterns and determine the abiotic conditions to which ecosystems are exposed [23]. Changes in these processes over time are likely to be reflected in community structure and can be recorded in the fossil record [24]–[26].

This study utilizes dinosaur taxa from several Late Jurassic (161–145 Mya) fossil localities from around the world. The Late Jurassic is notable for its extremely warm and equable climate, which was dominated by a monsoonal circulation pattern [27]–[30]. These extreme conditions played a large role in the distribution and diversity of Late Jurassic biota [31]. Furthermore, throughout the Jurassic many dinosaur clades diversified [32], leading to the evolution of many extreme morphologies characteristic of these groups. While often speculated upon, the ecological role of these adaptations remains poorly understood, particularly how these adaptations were integrated to form stable, operational communities. How were dinosaur-dominated communities structured and what role did climatic conditions play? It is well known that biological communities evolving under similar environmental conditions often contain convergent adaptations [33]–[36]. We therefore predict that dinosaur fossil assemblages falling under similar climatic conditions will exhibit convergent community structure irrespective of their individual phylogenic or biogeographic history.

## Materials and Methods

### Ecological Structure Analysis

The reconstruction of dinosaurs as living animals has greatly benefited from the increased emphasis on biomechanics and functional morphology as applied to fossil organisms. Use of biomechanical principals and a better understanding of the relationships between function and form substantially improve our ability to generate hypotheses about the behavior of extinct taxa [37]–[43]. These studies have led to many new insights about dinosaur paleoecology.

Traditionally, community analysis (as represented by fossil assemblages) uses indices of species richness [44] and/or taxonomic diversity indices (e.g., Simpson's Diversity Index). Species richness only analyzes the abundance of taxa at a site, and becomes extremely problematic when we lack tight control over the rate(s) of fossil accumulation at a site. In many cases, dinosaur fossil assemblages are a time-averaged collection representing a prolonged period of accumulation. Taxonomic diversity indices are more often used to determine the relative age of sites [45], [46] but may sometimes be used to make inferences about habitat as well [47]. However, on its own taxonomy is not sufficient to determine ecology. Different taxa may converge onto similar ecological niches, or alternatively closely related taxa may be ecologically diverse, especially when looking across broad spans of time. Thus, neither of these methods is sufficient for reconstructing the ecosystem represented at a fossil site.

Here we employ Ecological Structure Analysis (ESA), which uses functional morphology, to produce ecological profiles for different Late Jurassic localities. This method allows us to compare dinosaur assemblages, with varying taxonomic profiles and diversity, from a large number of localities. In applying ESA, taxa are classified using ecological criteria. Therefore, it is important to note that this is not a “taxon-free” method but merely a different way of classifying taxa. Reed [48]–[50] demonstrated that modern mammal communities located in different habitats differ significantly in the percentages of taxa found in trophic, locomotor, and body size categories (using both Kruskal-Wallis and the Mann-Whitney U tests), while those in similar habitats are more alike. She also demonstrated that the same ecological categories would correctly classify sites with different environments (using discriminant function analysis). Thus, it is possible to compare fossil mammal sites with modern ones and classify fossil localities according to habitat differences.

**Figure 1:**
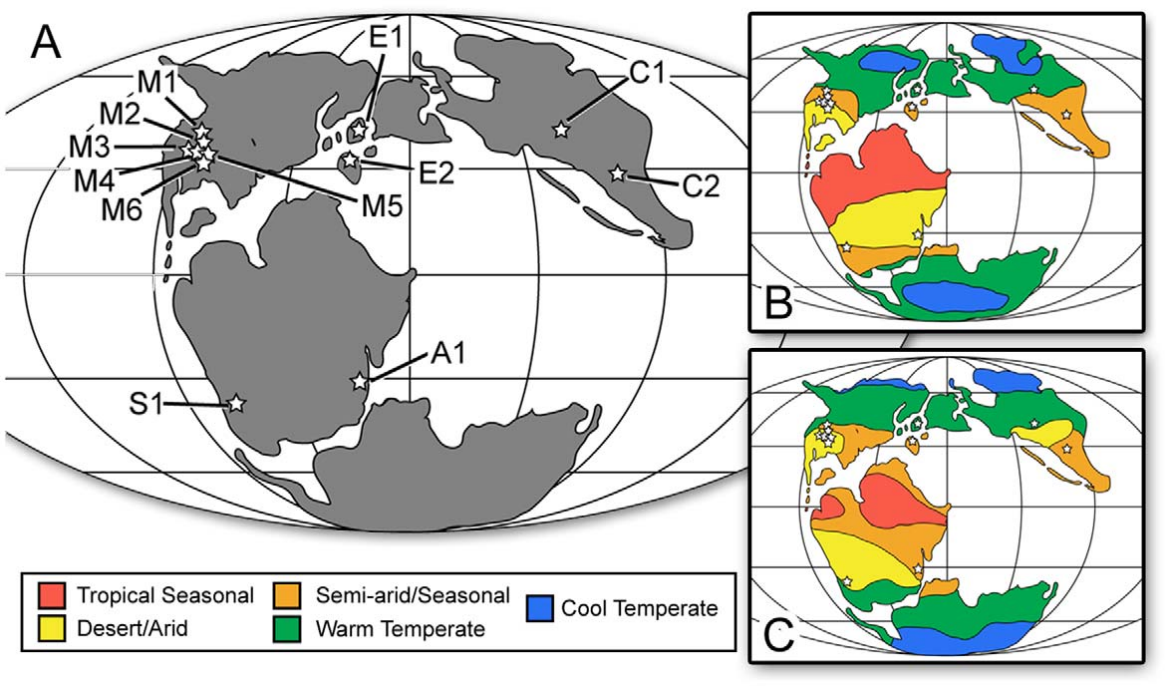
Late Jurassic paleogeographic map and reconstructed biomes. A) Positions of fossil assemblages (stars): A1 = Africa, C1–C2 = China, E1–E2 = Europe, M1–M6 = North America, and S1 = South America. Base map adapted from [27]. B) Biome reconstruction based on Rees et al. [27] model. C) Biome reconstruction based on Sellwood and Valdes [30] model.

For this study, ESA was applied in the following way. A database of fossil localities and taxon lists was compiled from the Paleobiology Database [51] and Weishampel et al. [52]. Each locality was plotted on a paleogeographic map (150 mya reconstruction, Mollweide projection) using the mapping function available on the Paleobiology Database. Geographically close localities or those forming natural clusters were grouped together to form composite assemblages (Figure 1). This was done to provide the necessary sample size for analysis. The twelve assemblages include (Table 1): six from North America (M1–M6), two from Europe (E1–E2), one from Africa (A1), one from South America (S1) and two from Asia (C1–C2). Complete data are available in Table S1.

**Table 1:** Location, biome assignments(s), and formations constituting each assemblage.

Assem.	Biome R^1^	Biome SV^2^	Region	Countries/States	Formation(s)
E1	2	1	Europe	England, France	Sables de Glos, Argiles d'Octeville, Marnes de Bléville, Kimmeridge Clay, Calcareous Grit, Corallian Oolite, Oxford Clay, Portland Stone
E2	2	2	Europe	Portugal, Spain	Villar del Arzobis, Alcobaça, Guimarota, Sobral, Amoreira-Porto Novo, Bombarral, Freixial, Louriñha
M1	2	3	North America	Wyoming, Montana, South Dakota	Morrison
M2	2	3	North America	Wyoming	Morrison
M3	3	3	North America	Colorado, Utah	Morrison
M4	3	3	North America	Colorado, Utah	Morrison
M5	3	3	North America	Colorado	Morrison
M6	3	3	North America	Colorado, New Mexico, Oklahoma	Morrison
C1	1	1	Asia	China (Xinjiang)	Shishugou, Kalazha
C2	2	2	Asia	China (Sichuan)	Shangshaximiao (Upper Shaximiao)
S1	2	3	South America	Chile, Argentina	Toqui, Cañadón Cálcereo
A1	2	3	Africa	Tanzania	Tendaguru

Biome Key: 1 = temperate, 2 = semi-arid/seasonally wet, 3 = desert/arid.

1 Climate model of Rees et al. [27].

2 Climate model of Sellwood and Valdes [30].

For each assemblage genera were counted only once and higher taxa (i.e., generically indeterminate remains) were counted only if the group was not represented by generic remains, unless published reports provided a reasonable case that the remains represent distinct taxa. Isolated teeth were used only if they were assigned to a taxon for which a specific size range is known. Each taxon was classified using three separate ecological categories: Body Size, Locomotor Mode, and Trophic Mode, themselves divided into multiple classes (Table 2). Classification within each category was determined using morphological correlates as determined from the fossil itself, published reconstructions (if available), and closely related taxa (especially if the taxa are fragmentary). While other categories may also be useful, we prefer these particular three because osteological and dental evidence is potentially available in the fossil record for all taxa studied. For each assemblage the percentage of taxa in each class was calculated, thus generating an ecological profile. It is important to note that this analysis deals with paleoecological diversity and assumes a degree of correspondence between the diversity and relative abundance of ecomorphs in an assemblage. A detailed description of all ecological categories and classes is given below.

**Table 2:** Description of ecological categories and constituent classes used in this study.

Ecological Category^1^	Characteristics	
**Trophic Mode**		**Code**
Carnivore	Eats meat	C
Herbivore	High browsing: above 5 meters	HH
	Intermediate browsing: up to 5 meters	HI
	Low browsing up to 2 meters	HL
	Ground foraging: up to 1 meter	HG
**Locomotor Mode**		
Terrestrial Quadruped	Moves quadrupedally on the ground	TQ
Terrestrial Biped	Moves bipedally on the ground	TB
Facultative Biped	Capable of quadru- and bipedal motion	TF
Terrestrial/Arboreal Biped	Moves and feeds on the ground and in trees	TA
**Body Size** 2		
Tiny	<1 kg	T
Very Small	1–10 kg	VS
Small	10–100 kg	S
Medium	100–1000 kg	M
Large	1000–10,000 kg	L
Very Large	>10,000 kg	VL

1 Ecological categories after [49].

2 Mass calculations from [63].

#### Body Size

Body size is perhaps the single most important ecological character. It not only influences lifestyle and behavior in an individual species, but is also responsible for driving ecosystem dynamics at higher levels of community organization. This occurs through the scaling of metabolic rate with body size, which influences population dynamics and species diversity, ultimately determining the flow of energy between trophic levels [53]–[57].

For individuals and species, body size can determine such ecologically meaningful characters as techniques of predator avoidance, the type of substrate an animal may utilize during locomotion [58], and the size of its home and day range [59]. In addition, body size generally affects overall mass and body proportions due to the mechanical constraints inherent in biological materials [60]. The forces that bodies of different size generate affect performance and therefore strategies in feeding, locomotion, and reproduction [61], [62]. In dinosaurs and other extinct organisms lacking modern analogs, many of these traits will remain difficult to determine directly from available fossil evidence. Use of body size estimates therefore can provide a first-order approximation of many important ecological characters when comparing taxa and assemblages.

Dinosaur body mass can be determined through estimate only. Each method has its own strengths and weaknesses, but discussion of these is outside the scope of this paper. More important is to provide a relative ranking of taxa even if absolute values are inaccurate. For continuity, we used body mass estimates based on Seebacher [63], which provides a wide list of taxa. For taxa not found on this list, we used the mass estimation equations given in the paper. We used only adult representatives where possible for mass estimation. Six size classes were used: Tiny (<1 kg), Very Small (1–10 kg), Small (10–100 kg), Medium (100–1000 kg), Large (1000–10 000 kg), and Very Large (>10 000 kg). Size classes are broad enough that expected body size variation among adults and estimate uncertainty is included for most taxa.

#### Trophic Mode

Trophic mode refers to the diet and food processing strategies of an animal, the two primary categories being carnivore and herbivore. Further divisions depend on our knowledge of the morphology and behavior of the group in question. Morphology of the teeth is most useful in determining what type of food the animal processed during its life. While it is true that the type of prey consumed and the manner in which it is captured varies considerably among carnivores, these specializations are often much harder to decipher from available fossil material. Nevertheless, among non-mammalian carnivores, teeth tend to be elongate, sharp, and pointed; sometimes laterally compressed with a blade-like edge. In addition, many non-mammalian carnivores have long, laterally compressed skulls and possess sharp, recurved claws that aid in prey capture, dispatch, and processing. In contrast, non-mammalian herbivores tend to possess shorter, blunter teeth with grinding, slicing, or shearing surfaces. Their skulls tend to be broad and short, and the limbs lack the kinds of claws typically seen in carnivores.

Due to the unknown nature of plant preference in herbivorous dinosaurs, we assign herbivores to categories of browse height rather than plant type. We assign only an upper browsing limit based on characters such as neck posture and limb length. Ground level forms the lower browsing limit. These classes are (from lowest to highest feeding height): Ground Forager (<1 m), Low Browser (<2 m), Intermediate Browser (<5 m), and High Browser (>5 m). The neck posture of sauropods remains contentious given their extreme and unusual morphology [38], [42]. However, general differences in vertical feeding position between sauropod taxa are supported by morphological, biomechanical, and tooth wear analysis [38], [42], [64], [65].

#### Locomotor Mode

We divide locomotor mode into two major categories, quadruped and biped. All locomotor modes used in this analysis are terrestrial in nature. Terrestrial quadrupeds are distinguished by having forelimbs that are closer in length to the hindlimbs and a robust radius locked with the ulna in a pronated position, increasing the stability of the front leg for weight-bearing during locomotion. In contrast, the forelimbs of bipeds are relatively shorter than the hind limbs. In addition, since the forelimbs are not necessary for weight-bearing the elbow joint is more mobile and the forelimb is not permanently locked in a pronated position. An additional division, facultative biped, was used for those taxa with intermediate axial and limb morphologies, such as shorter forelimbs capable of weight-bearing, suggesting that these taxa were capable of both bipedal and quadrupedal locomotion (e.g., some ornithopods) [66]–[68]. Some groups of dinosaurs evolved a quadrupedal stance secondarily, having descended from bipedal ancestors; however, these patterns in limb proportion and structure generally still hold.

The division Arboreal Biped was introduced because the locomotor behavior of some small maniraptorans and early birds (i.e, *Archaeopteryx*) is still debated [69]–[73]. For this analysis, we expect arboreal bipeds to have structures adapted for a combination of climbing, flight (gliding or active), and terrestrial locomotion, indicating they spent some amount of time both in trees and on the ground.

We acknowledge the great morphological variability in dinosaurs within the biped and quadruped categories, signaling important differences in locomotor strategy. More realistically, the locomotor strategies of taxa fall along a continuum, with discrete categories serving to delineate, rather than fully describe, how organisms move [74]. Again, further biomechanical work is necessary to determine how different limb and axial morphologies affected locomotor performance in dinosaurs. This work is currently underway and should lead to greater refinement of locomotor categories in the future [43], [75]–[78].

### Comparison with Late Jurassic Climate

Biomes were used over individual locality-level environmental reconstructions to observe how well independent climate data reflect ecological differences between assemblages. Biomes are characterized by different groups of plants, which help form the basic structure of every environment and are related to the individual climatic tolerance of each species. This structure plays an important role in directing the subsequent evolution of constituent species inhabiting these areas. Therefore, biomes provide an estimate of the general environmental and climatic conditions that prevailed over the regions where different fossil localities formed. Furthermore, like ecological categories, biomes can be extended and applied to other time periods because they in part rely on morphological categories for assigning plant taxa [27], [79].

Each assemblage was assigned a biome using reconstructions from Rees et al. [27] or Sellwood and Valdes [30]. The former is based primarily on the distribution and morphology of fossil plant taxa and the distribution of climate sensitive sediments (e.g., evaporites and coals), similar to methods used in delineating modern biomes. The latter is based on a general circulation model (GCM), delineating each biome from model-predicted temperature and precipitation patterns (Table 1). While generally similar, each reconstruction differs in their interpretation of certain dinosaur habitats and should therefore be considered when comparing the assemblages.

### Statistical Analyses

In order to compare ecological profiles, data were first arcsin transformed prior to analysis to approximate normality [80]. Similarity between assemblages was assessed with cluster analysis, utilizing the unweighted pair-group average (UPGMA) algorithm and Euclidean similarity measure. The stability of each node was assessed with a bootstrap test of 1000 replicates. In order to explore which ecological classes are most responsible for separating assemblages, a principal components analysis (PCA) was performed utilizing a covariance matrix. The number of significant components was determined using the eigenvalue bootstrapping method described in Peres-Neto et al. [81], in which we used the minimum recommended number of 1000 replicates. All analyses were run with PAST v 1.85 [82].

## Results

### Cluster Analysis

**Figure 2:**
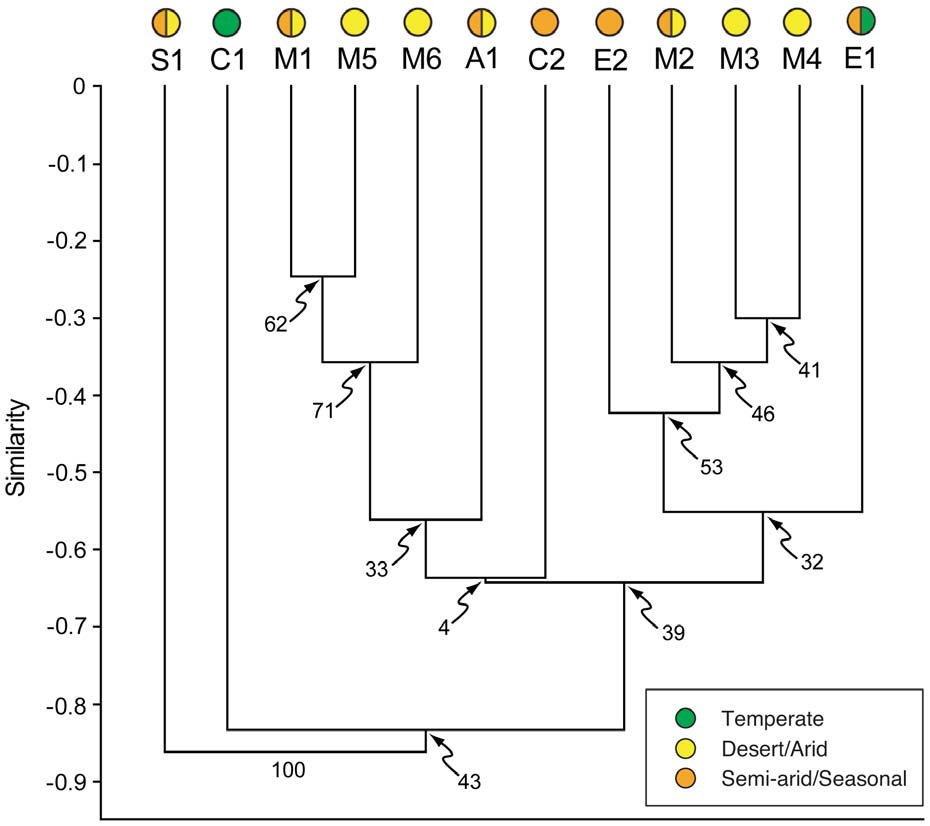
Results of cluster analysis (UPGMA with Euclidean similarity) for twelve Late Jurassic dinosaur fossil assemblages. Numbers indicate support for node (bootstrap: N = 1000). Labels refer to the fossil assemblage (see Table 1). Symbols denote the climate conditions assigned from the biome map. Two colors are shown if the biome assignment differs between the climate models.

Each assemblage exhibited a distinct ecological profile (Table 3). Cluster analysis separated the assemblages into several groups (Figure 2). S1 was found distinct from all other assemblages, while C1 was the next most distinct. The remaining ten assemblages are split into two groups, with the North American assemblages (M1–M6) split evenly between them. A1 and C2 are associated with one of these groups, while the other contains both E1 and E2. However, support for most nodes does not surpass 50%, except for those linking M1, M5, and M6 (73%) and E2, M2, M3, and M4 (52%). The relationship of C2 to either of the large nodes appears especially uncertain given its very low support (5%). Due to its lack of relationship with any other assemblage, S1 was excluded from further analysis.

**Table 3:** Percentages of taxa within ecological categories used in this study.

		Trophic Mode					Locomotor Mode				Body Size					
Assem.	Total Taxa	C	HH	HI	HL	HG	TQ	TB	TF	TA	T	VS	S	M	L	VL
E1	12	25.0	16.7	8.3	41.7	8.3	41.7	41.7	16.7	0.0	0.0	8.3	8.3	33.3	25.0	25.0
E2	24	37.5	12.5	25.0	20.8	4.2	50.0	41.7	4.2	4.2	4.2	12.5	8.3	16.7	29.2	29.2
M1	14	28.6	14.3	35.7	21.4	0.0	57.1	35.7	7.1	0.0	0.0	0.0	7.1	21.4	28.6	42.9
M2	22	36.4	9.1	27.3	18.2	9.1	45.5	50.0	4.5	0.0	0.0	9.1	22.7	18.2	13.6	36.4
M3	14	42.9	14.3	14.3	14.3	14.3	35.7	57.1	7.1	0.0	7.1	7.1	21.4	21.4	14.3	28.6
M4	20	40.0	10.0	20.0	25.0	5.0	45.0	50.0	5.0	0.0	5.0	5.0	15.0	25.0	20.0	30.0
M5	14	35.7	14.3	21.4	28.6	0.0	50.0	42.9	7.1	0.0	0.0	0.0	7.1	28.6	28.6	35.7
M6	10	30.0	20.0	30.0	20.0	0.0	60.0	30.0	10.0	0.0	0.0	0.0	0.0	20.0	30.0	50.0
C1	10	30.0	0.0	20.0	20.0	30.0	40.0	60.0	0.0	0.0	0.0	10.0	30.0	10.0	30.0	20.0
C2	11	27.3	9.1	27.3	27.3	9.1	63.6	36.4	0.0	0.0	0.0	9.1	0.0	27.3	27.3	36.4
S1	6	33.3	33.3	16.7	16.7	0.0	66.7	33.3	0.0	0.0	0.0	0.0	16.7	0.0	33.3	50.0
A1	11	45.5	9.1	27.3	18.2	0.0	45.5	54.5	0.0	0.0	0.0	0.0	18.2	36.4	18.2	27.3

Trophic Mode: C = carnivore, HH = high browser, HI = intermediate browser, HL = low browser, HG = ground foraging. Locomotor Mode: TQ = quadruped, TB = biped, TF = facultative biped, TA = arboreal biped. Body Size: VS = very small, S = small, M = medium, L = large, VL = very large. See text and Table 1 for explanation.

### Principal Components Analysis

**Figure 3:**
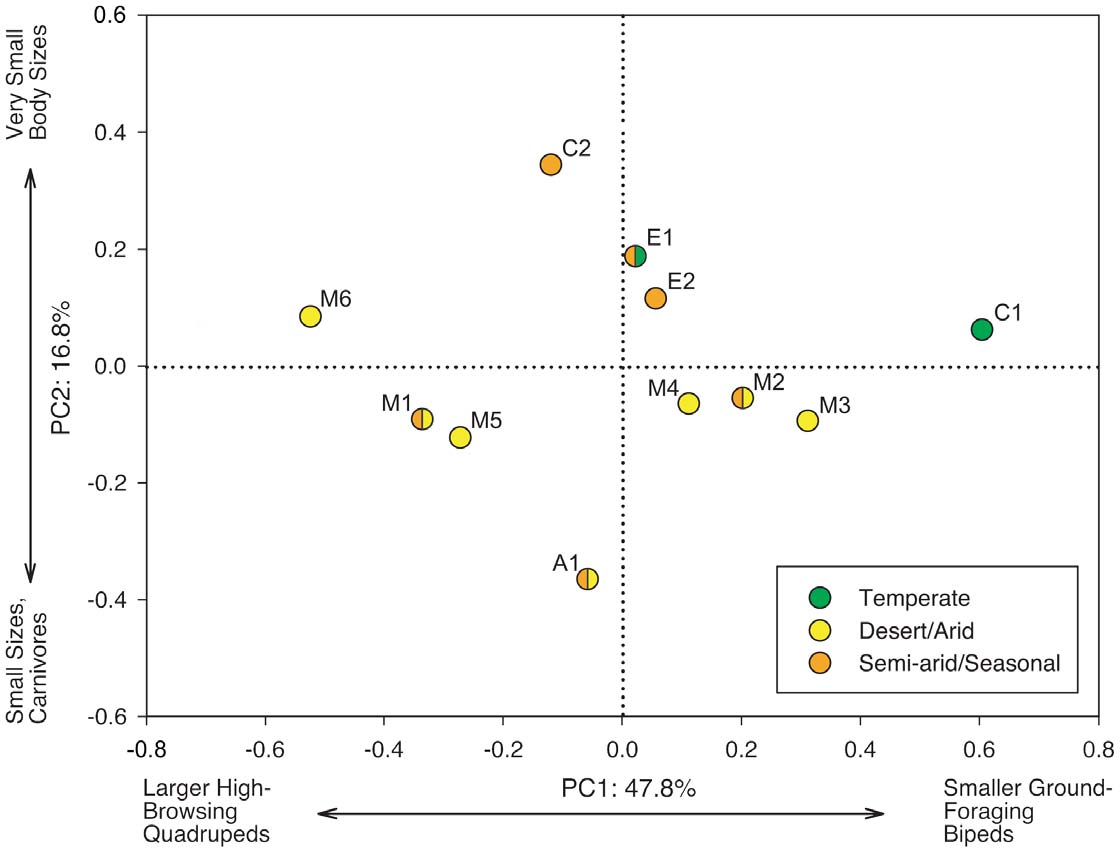
Results of Principal Components Analysis on twelve Late Jurassic dinosaur fossil assemblages. Symbols denote the climate conditions assigned from the biome map. Two colors are shown if the biome assignment differs between the climate models. PC 1 corresponds to differences in the proportion of herbivore body sizes and feeding strategies, while PC 2 follows other environmental, taphonomic, and historical differences between the assemblages.

Two significant principal components were recovered, which together account for 64.6% of the variance in the data (Figure 3). PC 1 accounts for 47.8% and PC 2 accounts for 16.8% of the variance, respectively. Positive values of PC 1 are associated with Ground Foragers, Small and Very Small sizes, and Bipeds, while negative values are associated with High Browsers, Very Large taxa, and Quadrupeds (Table 4). Positive values of PC 2 indicate a larger proportion of Very Small taxa while negative values suggest a greater proportion of Small taxa and Carnivores. The assemblages do not group tightly together but instead appear to fall along the continuum of PC 1, with members of the two major groups noted in the cluster analysis falling to either side of the origin. In order of increasing PC 1 score: M6, M1, M5, C2, A1, E1, E2, M4, M2, M3, and C1. Along PC 2 the greatest outliers are C2, E1, and A1. With the exception of assemblages M2, M3, and M4, each assemblage generally falls along PC 1 according to its biome. Assemblages with greater aridity have negative values, semi-arid assemblages remain near the origin, and assemblages representing moister environments are more positive.

**Table 4:** Variable correlation values for the two major principal component axes.

Variable	Code	PC Axis 1	PC Axis 2
Carnivore	C	0.239	−0.7485
High Browser	HH	−0.7404	−0.01778
Intermediate Browser	HI	−0.4834	−0.183
Low Browser	HL	−0.2177	0.4712
Ground Forager	HG	0.8879	0.4218
Quadruped	TQ	−0.7707	0.3876
Biped	TB	0.864	−0.467
Facultative Biped	TF	−0.3563	0.07475
Arboreal Biped	TA	0.05912	0.2034
Tiny	T	0.3478	−0.07956
Very Small	VS	0.7331	0.5727
Small	S	0.8023	−0.545
Medium	M	−0.4491	−0.2363
Large	L	−0.3843	0.4739
Very Large	VL	−0.8327	0.06462

## Discussion

### Relationship with Climate Reconstructions

Based on the correlations between ecological categories and principal components, general characteristics are ascribed to the biota and climatic conditions of each assemblage based on its position in the component plot. PC 1 represents climatic differences between the assemblages: negative scores indicate aridity and positive scores more temperate conditions. PC 2 is discussed below.

Ultimately, assemblages did not group together by biome as expected. This discrepancy is related to 1) the different climate reconstructions used and 2) small-scale environmental variation between the localities comprising each assemblage. The biome assignments for each assemblage from Rees et al. [27] and Sellwood and Valdes [30] largely agree. Key differences occur in regions that are transitional zones between adjacent biomes. The actual boundaries between biomes are gradational and much less distinct and likely migrated in response to long-term shifts in climate patterns. This mainly affects the assessment of assemblages M1, M2, E1and A1, which are alternatively assigned semi-arid or desert/arid conditions (Table 1). In many of the assemblages, published environmental reconstructions differ from their assigned biome and may help interpret the results. These discrepancies point to major differences in the way climate models and ecological data reconstruct environmental conditions. Therefore, the model-assigned designations may not conform to the modern conception of these environments and instead relate to relative differences specific to the equable climate of the Late Jurassic [Foster, pers. comm.]. The conditions assessed for each assemblage are briefly discussed below.

#### Europe (E1 and E2)

Throughout the Late Jurassic western Europe became progressively more arid with a strongly seasonal, Mediterranean-type climate [28], [83]–[86]. E1 likely represents a drier environment, making it more similar to E2, and in accordance with the Rees et al. [27] biome model. This is supported by both the cluster analysis and PCA results, which place E1 and E2 close to the middle of the continuum. Both assemblages are therefore considered to have had a semi-arid climate.

#### North America (M1–M6)

The Morrison Fm. is reconstructed as a seasonally dry, savannah-like environment; much moister than both models, which indicate greater aridity. In addition these assemblages do not consistently group together. The spatial extent and topography of the Morrison depositional basin sheds some light on this pattern. Recent work suggests two spatial gradients exist in the Morrison Fm.: a south to north, arid to temperate climate gradient and an east to west precipitation/drainage gradient [87], [88]. These conditions created a greater proportion of lakes and wetlands towards the center of the depositional basin [89]. This work supports the observed division between Morrison Fm. assemblages, with M3 as a possible exception. M3 is expected to group with “drier” Morrison assemblages due to its southwestern position, although this relationship may not be resolvable with the current arrangement of assemblages and/or using dinosaurs only. Therefore, assemblages M2, M3 (tentatively), and M4 are considered to have been semi-arid or more seasonally wet, while M1, M5, and M6 were likely more arid or strongly seasonal.

#### Asia (C1 and C2)

Climate reconstructions for many formations in China indicate semi-arid and seasonal conditions [90]–[92], however Hallam [28], [84], [86] reconstructs eastern Eurasia as being moister than the west. Each biome model places C1 as a temperate assemblage and C2 as seasonally semi-arid. Both analyses find C1 is quite different from the other assemblages, which could be indicative of a temperate climate, although this is unlikely. C1 occurs close to the boundary making its biome assignment tenuous at best. Sedimentary indicators of seasonality further refute the temperate nature of this assemblage [91], [92]. Tectonic uplift throughout the Jurassic increased seasonality throughout the region, which was not accounted for in either biome model. Therefore C1 was likely semi-arid, but perhaps less seasonal or experienced more intense moist periods than other areas. C2, on the other hand, lies between the two major groupings making it semi-arid and more seasonal than C1.

#### Africa (A1)

The Tendaguru Fm. has been reconstructed with a semi-arid climate with coastal influences that maintained somewhat higher moisture levels than seen inland [93], supporting the biome model of Sellwood and Valdes [30]. The intermediate position of A1 in both analyses agrees with the assignment of a semi-arid climate to this assemblage.

#### South America (S1)

The region surrounding S1 may have shared a similar coastal semi-arid climate [94], [95, Rauhut, pers. comm.], but the number of dinosaur fossils from this area remains too sparse to allow a full comparison here. We will have to wait until further dinosaur fossils from the area are described.

### Assemblage-Level Patterns

Results suggest that climatic and ecological factors played an important role in the distribution of Late Jurassic dinosaurs. In mammals, ecological preferences are often shared at the genus level or above [96], and the same was likely true of dinosaurs. The climatic and ecological similarity found here between North America, Europe, and southern Africa supports the biogeographic connections between these regions [5], [97], [98]. C2 shares relatively few taxa in common with the other semi-arid assemblages [5], [98], yet its general ecological similarity points to an ecosystem that evolved convergently under the same climatic conditions.

Generally, the faunas of the Morrison Fm. have been treated as a single unit, however it appears they were more ecologically diverse than previously thought, despite the relatively uniform distribution of dinosaur taxa [99], [100]. The ecological subdivisions present within the Morrison Fm. may also be related to its long history of intense study, which led to hundreds of described localities. This number is unmatched by the other assemblages. Future work may find further ecological subdivisions as more fossil localities are added in other regions.

C1, as the northernmost assemblage represented here, remains taxonomically and ecologically distinct. C1 may be like the central Morrison Fm. (M2–M4), maintaining greater moisture levels despite a seasonally semi-arid climate, or it may represent an entirely different semi-arid fauna that has yet to be encountered elsewhere.

PC2 appears related to assemblage-specific differences that contributed to the low bootstrap support in the cluster analysis. The wide distribution of scores among assemblages suggests PC2 cannot be directly attributed environmental variation. Instead, PC2 most likely represents some mixture of environmental, taphonomic, and sampling effects. For example, A1, E1, and C2 have high PC2 scores and also have a small number of taxa. Alternatively, this may be some relative measure of error in the spatial and/or temporal grouping of localities in an assemblage. Unfortunately the current database does not allow for more complex analysis, but the connection of PC2 to the ecology of these assemblages is certainly worthy of further inquiry.

### Relationship with Dinosaur Paleoecology

**Figure 4:**
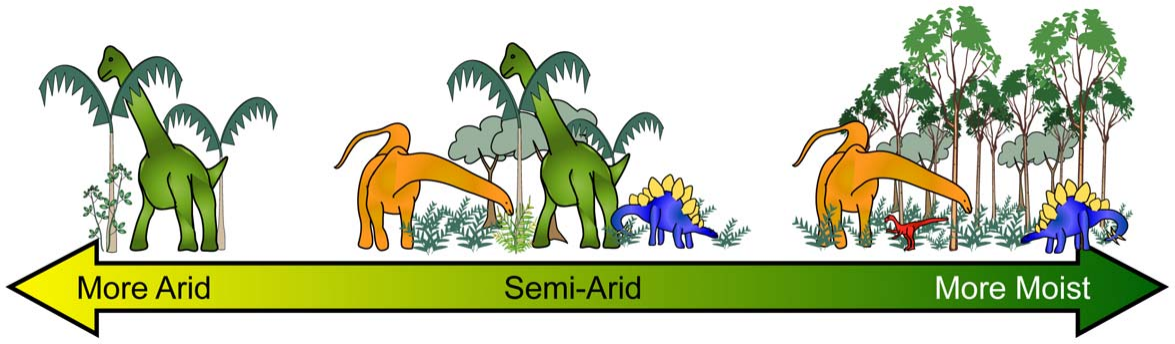
Schematic representation of variation in herbivore types and inferred habitat structure along PC 1. Relatively arid conditions occur to the left (negative values), where very large, high-browsing herbivores dominate among sparser foliage. Assemblages with semi-arid/seasonal conditions are towards the center (low values), which includes a greater diversity of feeding modes, including high, intermediate, and low browsers among increased ground cover. To the right are more moist conditions (positive values), where smaller, ground-foraging herbivores are more prevalent within a more densely vegetated environment. Carnivorous theropods appear largely independent of this pattern. Green = high browser, orange = intermediate browser, blue = low browser, red = ground forager.

The distribution of ecological categories varies accordingly with the proposed climatic differences between assemblages (Figure 4). Assemblages from more arid conditions (M1, M5, M6) tend to have a greater proportion of large-bodied taxa and lack anything smaller than 10 kg. High- and intermediate-browsing herbivorous sauropods and large carnivorous theropods such as *Allosaurus* dominate these assemblages. Smaller carnivores and ground-foraging herbivores are rare or absent. This pattern may reflect a lack of low-lying vegetation for a period of the season that restricted the numbers of smaller dinosaurs. Larger dinosaurs, on the other hand, were better able to cope with lower resource density and quality [101]–[104]. Assemblages representing semiarid or seasonally wet conditions usually contain representatives from each size class, though with no discernable decrease in the proportion of larger size classes. Ground Foragers are present as a larger portion of the herbivore population and High Browsers are less prevalent, indicating that more ground cover was likely available capable of supporting a greater diversity of herbivores. With increasingly moist conditions, assemblages exhibit greater proportions of Ground Foragers and Low- and Intermediate Browsers. In the extreme case of C1 High Browsers are conspicuously absent, reflecting perhaps a lack of suitable habitat or resources.

The environmental dependence of certain ecological categories demonstrates the importance of habitat structure in driving the relative abundance of dinosaur herbivore guilds. As observed in modern ecosystems, this is due to differences in habitat structure and resource availability. In the PCA results none of the herbivore classes are strongly positively correlated with each other. When the proportion of one herbivore class is high, the remaining classes are typically lower, reflecting the varying ecological roles each class plays in different environments or their differing habitat requirements. A similar trend is found among the herbivorous dinosaurs of the Morrison Formation [22], although this pattern occurs across different depositional environments instead of biomes.

Arid climates typically host sparse, open environments more suited for the largest animals. Small herbivores may suffer not only from a lack of food, but suitable cover from predators. Increasing moisture levels lead to more abundant growth, which inhibits the largest herbivores through changes to habitat structure and/or resource distribution, while becoming more favorable for smaller herbivores. The overall proportion of carnivores appears largely independent from environmental conditions although the absence of smaller predators in arid environments may be related to the lack of appropriately-sized prey and/or successful competitive exclusion by larger predators.

### Limitations of the Current Study

Despite the encouraging results, it is important to note some caveats. First, not all formations and regions have been equally explored. Historically, excavations have been conducted in North America and Europe more extensively than elsewhere. Spectacular discoveries from China in recent decades show that major strides in the number of vertebrate localities from a region can be accomplished rapidly. The attention now afforded to Africa and South America will hopefully yield similar results in the future.

The role of taphonomic factors presents the greatest uncertainty because its ultimate effect may depend on the scale of observation. Environment-specific taphonomic filtering may drive taxonomic variation between individual localities; aggregating the localities into assemblages minimizes this effect. However, at larger spatial scales climatic processes, especially the onset of arid conditions, affect vertebrate preservation [25], [31], [91], [105]. Under such conditions small size classes should be underrepresented in the fossil sample, potentially biasing the ecological profiles towards larger size classes with increasing aridity. The addition of vertebrate microfossil localities to an assemblage may help overcome this problem because these sites better reflect the community structure of the surrounding landscape, including both large and small taxa [106]. Dinosaur microfossil sites are found in the Morrison (M1–M4) and Camadas de Guimarota (E2) Formations, but are absent elsewhere [22], [97], [100]. Dinosaur microfossil remains usually consist of small theropod teeth, only some of which are assignable to useable taxa (see Materials and Methods and Table S1). The relationship between some assemblages change with removal of tooth taxa, but the climatic associations noted above remain more or less the same, indicating such localities are not necessary to assign climatic conditions but are useful is resolving ecological relationships among assemblages. Nevertheless, the role of microfossil localities and taphonomic filtering requires further scrutiny.

In addition to addressing taphonomic biases, it was necessary to group localities in order to achieve a minimum sample size for analysis. The relationships of assemblages with fewer than 10 taxa were found to be unresolvable, as in the case of S1. A small number of taxa skews the content of the categories since class data are calculated as a proportion of the total number of taxa and 10 is suggested here as a minimum sample for this type of study. While necessary, grouping spatially and stratigraphically distinct localities in this way increases the likelihood of including taxa and environments that never coexisted in life. Each assemblage therefore represents a coarse average of ecological conditions. Multiple studies have found overall taxonomic stability of the Morrison fauna through time [22], [99], [100]. The Tendaguru fauna of Africa was also similarly stable through the Late Jurassic [93]. The majority of formations included here lack such detailed biostratigraphic study. Paleoecological studies across a broad spectrum of scales, environments, and taxonomic groups have found that many past ecosystems maintained a stable structure over timescales of 100 ky to 2 my or more, despite major taxonomic turnovers or climatic events [107]–[113]. In this case, resolution of small-scale ecological differences are lost that may lead to interpretations of individual fossil localities disagreeing with the general results presented here.

A continuing challenge that requires more attention regards the assignment of ecological classes to dinosaurian taxa. In many cases the ecological niche of a dinosaur is still assessed using qualitative comparisons with living forms. Even with complex biomechanical models, very different interpretations of ecologically relevant morphological and behavioral reconstructions continue to arouse debate [114], [115]. This problem becomes more acute in taxa based on incomplete and fragmentary remains. Altering one or more classes for a single taxon has little effect, though more than these can alter the results. As always, new fossil finds may lead us to radically revise our view on the ecology of certain taxa. If multiple interpretations do exist separate analyses should be run using all combinations of interpretations to assess their effect on the results.

### Conclusions

Our study demonstrates that ESA is a useful tool for quantifying ecological differences between Late Jurassic dinosaur assemblages. The grouping of climatically similar assemblages supports the preservation of ecological structure at large scales and helped assess the accuracy of two different paleoclimate models. Ecological similarities are most likely related to differences in habitat structure due to variation in moisture availability; most important among these are the relative proportions of herbivore and body size classes in an assemblage. Not only can these proportions be used as additional climate indicators, but also may provide evidence of ecosystem convergence when comparing taxonomically distinct dinosaur localities. Although most Late Jurassic fossil localities come from relatively arid or semi-arid environments [31], there is a surprising amount of ecological variation that warrants further study.

A more comprehensive understanding of broad-scale ecological patterns is important in understanding the effect of climate patterns on dinosaur ecology and evolution. Perhaps more importantly, this study provides a framework for studying the long-term evolutionary dynamics of terrestrial communities related to climate change, major adaptive radiations, or evolutionary events (e.g., angiosperm evolution) and whether these events had a significant impact on the subsequent structure of vertebrate communities. These data can then be used to test hypotheses related to community formation and ecosystem function. A great deal of effort has been spent quantifying global taxonomic diversity levels through the Phanerozoic [116], [117], however these estimates tell us little about the ecological factors responsible for producing that diversity, nor how it was distributed on the surface [118].

## Supporting Information

Table S1 List of Late Jurassic dinosaur taxa and their assigned ecological categories used in this analysis, organized by assemblage. Diet: C = carnivore, HH = high browser, HI = intermediate browser, HL = low browser, HG = ground foraging. Locomotion: TQ = quadruped, TB = biped, TF = facultative biped, TA = arboreal biped. Body Mass: VS = very small, S = small, M = medium, L = large, VL = very large. See text and Table 1 for explanation. “PBDB Collection #” refers to the collection number of the specimen in the Paleobiology Database. Formations given in the table do not necessarily reflect all units in which the taxon is found. Data come from [51] and [52].
(0.05 MB XLS)
